# Reductions in Advanced Chronic Liver Disease Among People Who Inject Drugs: The ETHOS Engage Study

**DOI:** 10.1111/liv.70940

**Published:** 2026-10-30

**Authors:** Anna Conway, Heather Valerio, Jessie Payne, David Silk, Alison D. Marshall, Marianne Martinello, Shane Tillakeratne, Putu Novi Arfirsta Dharmayani, Andrew Milat, Janaki Amin, Phillip Read, Louisa Degenhardt, Annabelle Stevens, Jeremy Hayllar, Michael Christmass, Victoria Cock, Jessica Doumany, Jason Grebely, Gregory J. Dore, the ETHOS Engage Study Group, Amanda Erratt, Kyle Leadbeatter, Carla Treloar, Adrian Dunlop, Carolyn Murray, Mary Ellen Harrod, Mel Rhagnath, Clarke Scott, Vicky Cock, Jess Doumany, Dominic Mounsey, Maria Martinez, Amanda Erratt, Nadine Ezard, Craig Rodgers, David Reid, Carla Gorton, Jeremy Hayllar, Thao Lam, Adrian Dunlop, Prasun Datta, Alex Wade, Sally Spruce, Vicki Cock, Sarah Michelmore, Mark Cornwell, Krista Zohrab, Kate Salisbury, Patricia Collie, Craig Connelly, Angela Cooper, Mark Montebello, Chris Gough, Robert Cherry, Julie Dyer, Shikha Agrawal, Elke Press, Alice Wang, Teodora Zanesheva‐Karamanlieva, Eleanor Kessler, Nadine Horasak, Rhondda Lewis, Astrid Carthew, Daniel Morris, Kathy Donohue, Faith Bassett, Kathy Griffiths, Jason Dalla Lana, Sue Shin, Kylie Page, Julie Luff, Rebecca Hyland, Jane Booty, Todd McKillop, Connie Graf, Adele Hampson, Siyu Qian, Danielle Brown, Amber Lautier, Cate Curran, Peta McAlister, Peter Warburton, Rachel Pollard, Daniel Pekin‐Stores, Jessica Rees, Danielle Gaudiosi, Tammy Raikko, Martina Camitz, Corrie Brandsma‐McGrath, Emma Harkins, Nicole Stuart, Coby‐Lea Chalker, Carly Harvey, Edwin Christopher, David Khalil, Kerry McMorrow, Melissa Wellman, Carina Burns, Ravina Raidu, Kylie Stolzenhein, Wanda Brabender, Kelly Somes, Gary Keogh, Isabella Conwell, Nargis Abram, Rick Turner, Stuart Larter, Fiona Goodberg, Jennifer Luksza, Meryem Jefferies, Michelle Hall, Susan Hazelwood, Charlotte Ismay, Antoni Pazeski, Bettina Sweetman, Michelle Gallagher, Cecily Willis, Amanda Brown, Belinda McClurg, Cherie Mincham, Kali Barlow, Anita Hoskins, Laura Johnson, Courtney Priestley, Amie Johns, Shane Moore, Kerrie‐Ann Lahrs, Julie Markham, Jacky Talmet, Sandy Dunn, Amanda Mitchell, Lauren Bright, May Lee, Alcohol Services, Bronwyn Gordon, Blair Brewerton, Michael Wallace, Andrew McKinnon, Fionnuala Smyth, Lisa Snell, Elizabeth Laing, Martin Clark, Justin Dorigo, Louise Carman, Toni Hamilton, Ching Tan, Deidre O'Shea, Brent Fergusson, Bonny Puszka, Gai Duncan, Fiona Baker, Jayde Walsh, Leeann Walsh, Hannah Cook, Natasha Nikolic, Louise Hughes, Julia Bitmead, Dean Wang, Kareen Tait, Sav Gollapally, Cáitleen Moloney, Sarah Ahmed

**Affiliations:** ^1^ The Kirby Institute University of New South Wales Sydney New South Wales Australia; ^2^ Centre for Social Research in Health University of New South Wales Sydney New South Wales Australia; ^3^ Centre for Epidemiology and Evidence NSW Health Sydney New South Wales Australia; ^4^ Department of Health Systems and Populations Macquarie University Sydney New South Wales Australia; ^5^ Epidemiology and Systems Branch NSW Health Sydney New South Wales Australia; ^6^ Kirketon Road Centre Sydney New South Wales Australia; ^7^ National Drug and Alcohol Research Centre University of New South Wales Sydney New South Wales Australia; ^8^ Population Health Strategy & Performance NSW Health Sydney New South Wales Australia; ^9^ Alcohol and Drug Service, Metro North Mental Health Metro North Hospital and Health Service Brisbane Queensland Australia; ^10^ Next Step Community Alcohol and Drug Service Perth Western Australia Australia; ^11^ National Drug Research Institute Curtin University Perth Western Australia Australia; ^12^ Drug and Alcohol Services South Australia Adelaide South Australia Australia; ^13^ Australian Injecting and Illicit Drug Users League (AIVL) Canberra Australia

## Abstract

**Background and Aims:**

Liver disease is a significant risk for people who inject drugs due to the high burden of hepatitis C virus (HCV). There is limited data on the impact of the direct‐acting antiviral (DAA) therapy era on liver disease among people who inject drugs. This study evaluated baseline and longitudinal liver disease in a national cohort of people who inject drugs recruited over three Waves: 2018–2019, 2019–2021, and 2023–2025.

**Methods:**

The ETHOS Engage Study is an observational cohort of people who inject drugs attending drug treatment clinics and needle and syringe programs in Australia. Participants completed a questionnaire and underwent point‐of‐care HCV RNA testing (Xpert HCV Viral Load Fingerstick) and liver stiffness measurement (LSM) by vibration‐controlled transient hepatic elastography (FibroScan). Advanced chronic liver disease was defined by LSM of 10 kPa or above.

**Results:**

Among all enrolments with a valid LSM (*n* = 3126, 79% recruited at drug treatment clinics), 11% had advanced chronic liver disease, decreasing from 12.0% (154/1287) in 2018–2019 to 7.9% (66/839) in 2019–2021, and 6.4% (62/967) in 2023–2025. In adjusted analyses, participants recruited between 2019–2021 and 2023–2025 were less likely to have advanced chronic liver disease (vs. 2018–2019; aOR 0.66, 95% CI: 0.48–0.90 and aOR 0.56, 95% CI: 0.40–0.77, respectively). Current HCV infection declined from 24% in 2018–2019 to 10% in 2023–2025. Among participants with LSM in more than one wave and current HCV infection at first visit and no current HCV infection at second visit (*n* = 44), advanced chronic liver disease declined from 19% to 5%.

**Conclusions:**

Unrestricted access to DAA therapies in Australia has been associated with reduced liver disease burden among people who inject drugs. Monitoring of liver health in this population is important, given potential ageing and non‐HCV liver disease impacts.

## Highlights

- Advanced chronic liver disease decreased among people who inject drugs across three distinct recruitment waves.
- Unrestricted access to hepatitis C treatment and subsequent declines in infection has likely contributed toward this reduction.
- Ongoing follow up may be required to prevent liver disease in groups experiencing increased risk in the latest recruitment wave.

## Lay Summary

We observed a reduction in the burden of advanced chronic liver disease among people who inject drugs in Australia, likely due to unrestricted access to highly curative hepatitis C treatment, and subsequent increases in treatment uptake, and decreases in prevalence of infection. While encouraging, continued monitoring of liver health in this population is important, given ongoing liver disease risks outside of hepatitis C.

## 1 Introduction

Liver disease is a leading cause of morbidity and premature mortality, contributing to an estimated two million deaths each year globally [1]. Hepatitis C virus (HCV) can lead to progressive liver damage, resulting in cirrhosis and associated complications [2]. Liver disease is a major health issue for people who inject drugs due to high HCV prevalence [3]. In 2015, the World Health Organization set global targets to eliminate HCV as a public health threat by 2030 [4]. Achieving those targets is an important step toward reducing the burden of cirrhosis and hepatocellular carcinoma among people who inject drugs [5, 6, 7]. There is limited data on the impact of the direct‐acting antiviral (DAA) therapy era on liver disease among people who inject drugs.

Vibration‐controlled transient elastography is an important tool for liver stiffness measurement (LSM), avoiding invasive liver biopsies, and is highly acceptable among people who inject drugs [8]. This technology can evaluate the liver health impact of HCV elimination strategies among key populations. Population‐based data linkage studies have evidenced the impact of increasing DAA therapy uptake on hospitalisations due to cirrhosis and liver‐related deaths [9, 10, 11, 12, 13]. These studies often lack behavioural data, lack data on liver stiffness, or are limited to hospital‐based cohorts with inherent selection biases. Community‐based studies using transient elastography have explored liver disease among people who inject drugs [14, 15] and demonstrated the impact of DAA therapy on cirrhosis [16] but have not considered changes to liver health over time. Repeated bio‐behavioural surveys which collect HCV status and LSM can provide insights into the impact of DAA therapy on liver health of people who inject drugs.

This study evaluated baseline and longitudinal LSM in a national cohort of people who inject drugs recruited over three Waves: 2018–2019, 2019–2021, and 2023–2025, covering an era of unrestricted access to DAA therapy in Australia. Analyses include factors associated with advanced chronic liver disease, change in cross‐sectional prevalence of advanced chronic liver disease between waves, and longitudinal change in advanced chronic liver disease among those with current HCV infection at first enrolment and liver disease assessments in more than one Wave.

## 2 Methods

### 2.1 Data Sources

ETHOS Engage is an observational cohort study. Participants were enrolled across three recruitment waves. During Wave 1 (May 2018–September 2019) recruitment occurred in 25 sites, including drug treatment clinics (*n* = 21) and needle and syringe programs (NSPs) (*n* = 4). During Wave 2 (November 2019–June 2021), recruitment occurred in 21 of the 25 sites which participated in Wave 1, including drug treatment clinics (*n* = 19) and NSPs (*n* = 2). During Wave 3 (May 2023–Jan 2025), recruitment occurred in 28 sites (drug treatment clinics, *n* = 24; NSPs, *n* = 4) of which 21 participated in a previous Wave. Participants enrolled in each wave were identified by two‐by‐two name code (first two letters of first name and last name) and date of birth, enabling identification of those enrolled in more than one wave.

Inclusion criteria were informed consent, age ≥ 18 years, and injecting drug use, either within the previous six months or lifetime history and current opioid agonist treatment (OAT). Due to contraindications with vibration‐controlled transient hepatic elastography (via FibroScan, Echosens, Paris, France), people who were pregnant were excluded from Wave 1. The study protocol was amended for Wave 2 and Wave 3 to include pregnant participants (FibroScan withheld). For cross‐sectional analyses of each Wave, all participants in the Wave were included. For longitudinal analyses, the first and last enrolment were used for participants who participated in more than one Wave. The initial study protocol and amendments were approved by the Human Research Ethics Committees at St Vincent's Hospital, Sydney (HREC Ref: HREC/17/SVH/113) and the Aboriginal Health and Medical Research Council (HREC Ref: 1279/17).

### 2.2 Procedures

ETHOS Engage procedures have been previously described [17, 18]. ETHOS Engage was advertised preceding recruitment with posters, cards distributed with injecting equipment, and by word of mouth. Recruitment spanned 1–5 days per site and included a team of peers, university staff, and clinic personnel.

Campaign days were run in multiple stages. Participants provided 100 μL finger‐stick capillary whole‐blood to test for HCV RNA using the point‐of‐care Xpert HCV Viral Load Fingerstick Assay (Cepheid, Sunnyvale, United States; lower limit of quantification 100 IU/mL, upper limit of quantification 10^8^ log10 IU/mL) [19]. Participants then self‐completed a computer tablet‐based questionnaire collecting demographics, behavioural risk, and HCV history data. LSM was determined using FibroScan with either M probe or XL probe. An XL probe was not available in Wave 1, was used in Wave 2 when sites had one available (18/21 sites) and was supplied by ETHOS Engage at all sites in Wave 3. All participants underwent FibroScan unless they were pregnant or opted out. Participants underwent a consultation with appropriate clinical staff. All participants were compensated (AUD$30 gift voucher in Wave 1 and Wave 2, AUD$50 in Wave 3).

### 2.3 Outcomes at Enrolment Baseline

The primary measure for this study was liver disease stage based on LSM from FibroScan and using the Baveno VII rule‐of‐five cut offs: < 5, 5–9.9, 10–14.9, 15–19.9, 20–24.9, ≥ 25 kPa [20]. The primary outcome was proportion and predictors of advanced chronic liver disease (≥ 10 kPa). LSM from FibroScan was considered valid if it met all the following criteria: 10 or more readings, reading validity of 60% or more, and interquartile range to median ratio of 0.30 or less (if LSM was > 7.0 kPa).

### 2.4 Statistical Analysis

Logistic regression was used to assess factors associated with (1) advanced chronic liver disease (≥ 10 kPa) overall and (2) advanced chronic liver disease among those enrolled in the most recent recruitment Wave. For those who participated in more than one wave, all enrolments were included.

For the longitudinal analysis of participants enrolled in more than one Wave, the distribution of liver disease at first and second visits was evaluated by HCV status sub‐population: (1) never HCV infected; (2) spontaneous HCV clearance or treatment‐induced HCV clearance; (3) current HCV infection. In addition, for participants with current HCV infection at first visit, analyses were undertaken stratified by HCV status at second visit: (a) current HCV infection to HCV clearance; (b) current HCV infection to current HCV infection.

Demographic and behavioural factors hypothesized to be associated with advanced chronic liver disease were determined using previously published ETHOS Engage Wave 1 and Wave 2 results [17, 18] and include: (i) age, (ii) gender (male, female, other [non‐binary/transgender/other]), (iii) Aboriginal or Torres Strait Islander identification, (iv) homelessness, (v) OAT status (never, past, within the last month/current), (vi) incarceration history (never, history only [not recent], recent [previous 12 months in Wave 1 and previous six months in Wave 2 and Wave 3]), (vii) recency and frequency of injecting drug use (> 1 year ago, within the previous 1–12 months, within the previous month < daily, and ≥ daily), (viii) hazardous alcohol use (based on AUDIT‐C alcohol screening questions) based on frequency and quantity of alcohol consumption in last year, providing a score assigning participant to four possible categories; low/no use, mild misuse, moderate misuse, severe misuse [21], (ix) recruitment Wave (Wave 1, May 2018–September 2019; Wave 2, November 2019–June 2021; Wave 3, May 2023–Jan 2025).

Association between demographic and behavioural factors and advanced chronic liver disease was analysed in unadjusted analyses. Variables which had significance ≤ 0.20 at the unadjusted level or known clinical significance were considered for adjusted models. All analyses were conducted using Stata 17.0 (StataCorp, College Station, TX, USA).

## 3 Results

### 3.1 Sample Characteristics

A total of 3093 unique participants had valid LSM at their first point of contact, including 1287 enrolled during 2018–2019 (Wave 1), 839 in 2019–2021 (Wave 2), and 967 in 2023–2025 (Wave 3) (Table 1). Participants with no valid LSM (*n* = 472) included those without an attempted LSM (participant refusal, 5%; obesity, 13%; pregnancy, 0.5%; participant discomfort/injury/mobility issue, 4%), and those with an invalid result (78%). Among those with an attempted LSM (*n* = 3378) there were 8% with an invalid result. Of all unique participants with a valid LSM, 67% were men, median age was 44 years, 68% were receiving OAT, and 67% had injected drugs in the last month. For alcohol use, 61% reported low/no alcohol use, 10% mild misuse, 19% moderate misuse, and 11% severe misuse (Table 1). Based on HCV RNA testing, 18% had current HCV infection (4% previously treated and 14% not previously treated) (Table 1). Among individuals with valid LSM and ever eligible for HCV treatment (HCV RNA detectable or ever received treatment), 72% (1106/1525) self‐reported history of HCV treatment. Treatment coverage increased from 64% (448/696) in Wave 1, to 71% (285/398) in Wave 2, and 87% (373/431) in Wave 3.

**TABLE 1:** Baseline characteristics among unique ETHOS Engage participants with available liver stiffness measurement.

Characteristic	Total known LSM result, *n* (% col)
Total	3093 (100%)
Age at enrolment	
18–29	278 (9%)
30–39	788 (25%)
40–49	1167 (38%)
≥ 50	860 (28%)
Gender	
Men	2081 (67%)
Women	986 (32%)
Other	26 (1%)
Aboriginal or Torres Strait Islander	
No	2322 (75%)
Yes	771 (25%)
Homeless	
No	2725 (88%)
Yes	368 (12%)
OAT status	
Never	511 (17%)
Past	469 (15%)
Current	2113 (68%)
Incarceration history	
Never	982 (32%)
History only	1542 (50%)
Recent	569 (18%)
Recency of injecting	
> 12 months	376 (12%)
Within 1–12 months	630 (20%)
Within last month, < daily	1026 (33%)
Within last month, ≥ daily	1061 (34%)
AUDIT‐C alcohol use	
Low/no use	1859 (61%)
Mild misuse	299 (10%)
Moderate misuse	572 (19%)
Severe misuse	337 (11%)
HCV status	
Uninfected	986 (32%)
Spontaneous clearance	498 (16%)
Treatment‐induced clearance	979 (32%)
Current infection, previously treated	127 (4%)
Current infection, not previously treated	419 (14%)
Recruitment wave	
Wave 1 (2018–2019)	1287 (42%)
Wave 2 (2019–2021)	839 (27%)
Wave 3 (2023–2025)	967 (31%)

### 3.2 Liver Disease Distribution

Among participants with a valid LSM (*n* = 3093), 43.4% were < 5 kPa, 48.9% 5–9.9 kPa, 5.2% 10–14.9 kPa, 1.5% 15–19.9 kPa, 0.6% 20–24.9 kPa, and 1.5% kPa ≥ 25, indicative of clinically significant portal hypertension and high risk of end‐stage liver disease complications (Figure 1). Median LSM score was 5.5 kPa in Wave 1 (2018–2019), 5.0 kPa in Wave 2 (2019–2021), and 5.2 kPa in Wave 3 (2023–2025). Overall, 9.1% had advanced chronic liver disease (≥ 10 kPa) (Figure 1). Advanced chronic liver disease (≥ 10 kPa) decreased from 12.0% (154/1287) in 2018–2019 to 7.9% (66/839) in 2019–2021 and 6.4% (62/967) in 2023–2025. Advanced chronic liver disease was present in 1.1% of participants aged 18–29 years, compared to 14.4% in those aged ≥ 50 years (Table 2).

**FIGURE 1:**
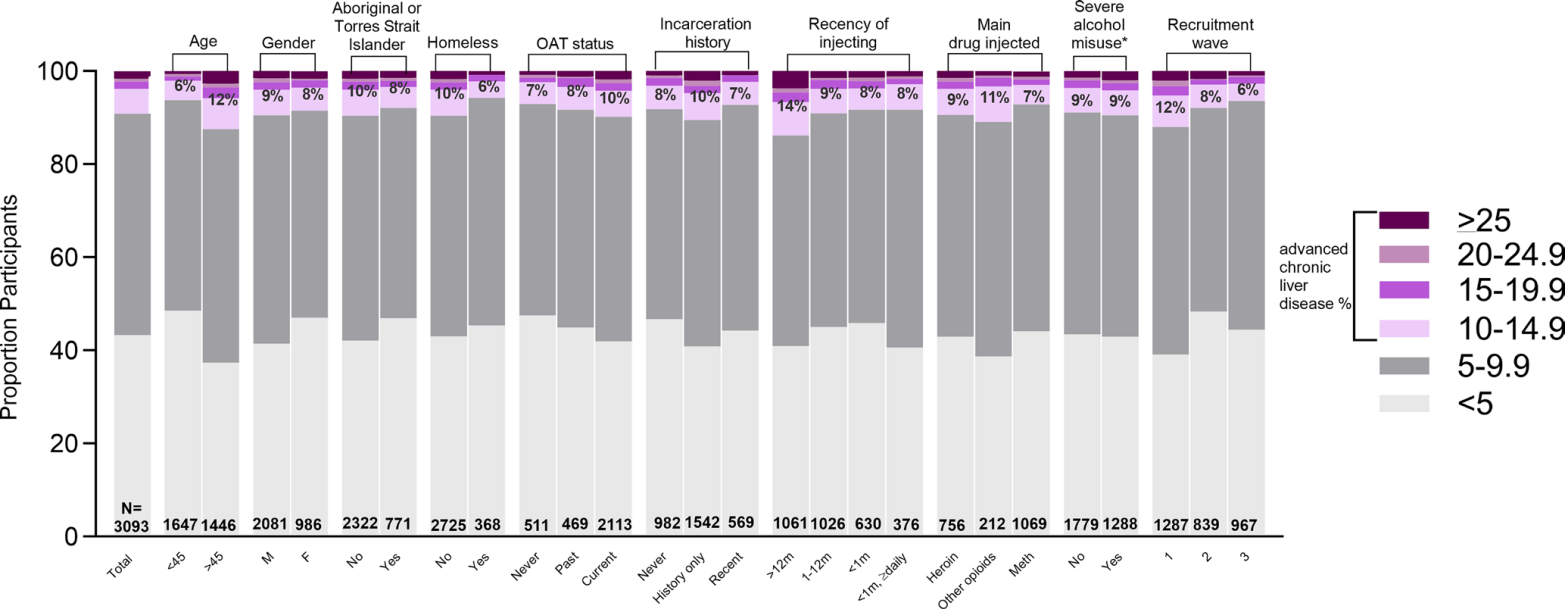
Proportion with advanced chronic liver disease among unique ETHOS Engage participants with available liver stiffness measurement.

**TABLE 2:** Factors associated with advanced chronic liver disease among unique ETHOS Engage participants with available liver stiffness measurement.

Characteristic	Advanced liver disease, *n* (% row)	OR (95% CI)	aOR (95% CI)
Total	282 (9.1%)		
Age at enrolment			
18–29	3 (1.1%)	0.06 (0.02–0.21)	0.08 (0.03–0.27)
30–39	50 (6.4%)	0.40 (0.29–0.57)	0.40 (0.28–0.57)
40–49	105 (9.0%)	0.59 (0.45–0.77)	0.59 (0.44–0.79)
≥ 50	124 (14.4%)		
Gender			
Men	197 (9.5%)		
Women	83 (8.4%)	0.88 (0.67–1.15)	0.93 (0.70–1.25)
Other	2 (8.7%)	0.80 (0.19–3.40)	0.97 (0.21–4.34)
Aboriginal or Torres Strait Islander			
No	221 (9.5%)		
Yes	61 (7.9%)	0.82 (0.61–1.10)	0.96 (0.70–1.31)
Homeless			
No	261 (9.6%)		
Yes	21 (5.7%)	0.57 (0.36–0.90)	0.63 (0.39–1.02)
OAT status			
Never	36 (7.1%)		
Past	39 (8.3%)	1.20 (0.75–1.92)	0.93 (0.56–1.53)
Current	207 (9.8%)	1.43 (0.99–2.07)	0.93 (0.62–1.40)
Incarceration history			
Never	80 (8.2%)		
History only	161 (10.4%)	1.31 (0.99–1.74)	1.03 (0.76–1.40)
Recent	41 (7.2%)	0.88 (0.59–1.30)	0.87 (0.57–1.34)
Recency of injecting			
> 12 months	52 (14.8%)		
Within 1–12 months	57 (9.1%)	0.62 (0.42–0.92)	0.60 (0.39–0.92)
Within last month, < daily	85 (8.3%)	0.56 (0.39–0.81)	0.54 (0.36–0.80)
Within last month, ≥ daily	88 (8.3%)	0.56 (0.39–0.81)	0.58 (0.39–0.88)
AUDIT‐C alcohol use			
Low/no use	167 (9.0%)		
Mild misuse	23 (7.7%)	0.84 (0.54–1.33)	
Moderate misuse	55 (9.6%)	1.08 (0.78–1.48)	
Severe misuse	35 (10.4%)	1.17 (0.80–1.72)	
HCV status			
Uninfected	43 (4.4%)		
Spontaneous clearance	40 (8.0%)	1.92 (1.23–2.99)	1.51 (0.94–2.42)
Treatment‐induced clearance	97 (9.9%)	2.41 (1.66–3.49)	1.80 (1.21–2.68)
Current infection, previously treated	25 (19.7%)	5.38 (3.15–9.16)	5.08 (2.88–8.94)
Current infection, not previously treated	72 (17.2%)	4.55 (3.06–6.77)	3.84 (2.52–5.85)
Recruitment wave			
Wave 1 (2018–2019)	154 (12.0%)		
Wave 2 (2019–2021)	66 (7.9%)	0.63 (0.46–0.85)	0.66 (0.48–0.90)
Wave 3 (2023–2025)	62 (6.4%)	0.50 (0.37–0.69)	0.56 (0.40–0.77)

### 3.3 Factors Associated With Advanced Chronic Liver Disease Across All Waves

In adjusted analyses, participants recruited in 2019–2021 (Wave 2) and 2023–2025 (Wave 3) were less likely to have advanced chronic liver disease (vs. 2018–2019 [Wave 1]; aOR 0.66, 95% CI: 0.48–0.90 and aOR 0.56, 95% CI: 0.40–0.77, respectively). Other factors associated with advanced chronic liver disease were age (vs. age ≥ 50, age 18–29 aOR 0.08, 95% CI: 0.03–0.27, age 30–39 aOR 0.40, 95% CI: 0.28–0.57, age 40–49 aOR 0.59, 95% CI: 0.44–0.79), and HCV status (vs. uninfected; treatment induced clearance: aOR 1.80, 95% CI: 1.21–2.68; currently infected and previously treated: aOR 5.08, 95% CI: 2.88–8.94; currently infected and no previous treatment: aOR 3.84, 95% CI: 2.52–5.85) (Table 2).

### 3.4 Factors Associated With Advanced Chronic Liver Disease Among Participants Ever HCV Infected

Among participants with a valid LSM and ever HCV infected (*n* = 2023), advanced chronic liver disease was present in 11.6% (*n* = 234). Advanced liver disease was 8.0% in people with spontaneous clearance, 9.9% in people with treatment‐induced clearance, 19.7% in people with current HCV infection who were previously treated, and 17.2% in people with current HCV infection who were not previously treated.

In adjusted analyses, participants recruited in 2019–2021 (Wave 2) and 2023–2025 (Wave 3) were less likely to have advanced chronic liver disease (vs. 2018–2019 [Wave 1]; aOR 0.66, 95% CI: 0.46–0.94 and aOR 0.61, 95% CI: 0.43–0.88, respectively). Other factors associated with advanced chronic liver disease were age (vs. age ≥ 50, age 18–29 aOR 0.10, 95% CI: 0.02–0.42, age 30–39 aOR 0.41, 95% CI: 0.27–0.61, age 40–49 aOR 0.55, 95% CI: 0.40–0.76), alcohol use (vs. low/no use; severe misuse: aOR 1.66, 95% CI: 1.07–2.59) and HCV status (vs. spontaneously cleared; currently infected and previously treated: aOR 3.45, 95% CI: 1.96–6.05; currently infected and no previous treatment: aOR 2.49 95% CI: 1.62–3.83) (Table S1).

### 3.5 Factors Associated With Advanced Chronic Liver Disease in Wave 3 (2023–2025)

Among participants with a valid LSM in the most recent Wave 3 (2023–2025) (*n* = 957), advanced chronic liver disease was present in 6.2% (*n* = 62). Advanced chronic liver disease was present in 3.2% in people aged 30–39, 5.3% in people aged 40–49, and 10.4% in people aged ≥ 50 (Table 3).

**TABLE 3:** Factors associated with advanced chronic liver disease among ETHOS Engage participants in Wave 3 with available liver stiffness measurement.

Characteristic	Total known LSM result, *n* (% col)	Advanced liver disease, *n* (% row)	OR (95% CI)	aOR (95% CI)
Total	1167 (100%)	72 (6.2%)		
Age at enrolment				
18–29	100 (9%)	0 (0.0%)		
30–39	217 (19%)	7 (3.2%)	0.29 (0.13–0.65)	0.38 (0.16–0.89)
40–49	456 (39%)	24 (5.3%)	0.48 (0.28–0.81)	0.51 (0.30–0.88)
≥ 50	394 (34%)	41 (10.4%)		
Gender				
Men	794 (68%)	40 (5.0%)		
Women	361 (31%)	32 (8.9%)	1.83 (1.13–2.97)	1.82 (1.07–3.09)
Other	12 (1%)	0 (0.0%)		
Aboriginal or Torres Strait Islander				
No	818 (70%)	53 (6.5%)		
Yes	349 (30%)	19 (5.4%)	0.83 (0.48–1.43)	
Homeless				
No	1025 (88%)	69 (6.7%)		
Yes	142 (12%)	3 (2.1%)	0.30 (0.09–0.96)	0.42 (0.13–1.37)
OAT status				
Never	191 (16%)	13 (6.8%)		
Past	225 (19%)	13 (5.8%)	0.84 (0.38–1.86)	
Current	751 (64%)	46 (6.1%)	0.89 (0.47–1.69)	
Incarceration history				
Never	353 (30%)	22 (6.2%)		
History only	621 (53%)	46 (7.4%)	1.20 (0.71–2.04)	1.10 (0.61–1.99)
Recent	193 (17%)	4 (2.1%)	0.32 (0.11–0.94)	0.49 (0.16–1.54)
Recency of injecting				
> 12 months	121 (10%)	12 (9.9%)		
Within 1–12 months	233 (20%)	17 (7.3%)	0.71 (0.33–1.55)	0.68 (0.31–1.53)
Within last month, < daily	339 (29%)	21 (6.2%)	0.60 (0.29–1.26)	0.62 (0.28–1.34)
Within last month, ≥ daily	474 (41%)	22 (4.6%)	0.44 (0.21–0.92)	0.46 (0.21–0.98)
AUDIT‐C alcohol use				
Low/no use	715 (61%)	41 (5.7%)		
Mild misuse	118 (10%)	5 (4.2%)	0.73 (0.28–1.88)	
Moderate misuse	193 (17%)	15 (7.8%)	1.39 (0.75–2.56)	
Severe misuse	129 (11%)	11 (8.5%)	1.53 (0.77–3.07)	
HCV status				
Uninfected	415 (36%)	13 (3.1%)		
Spontaneous clearance	188 (16%)	15 (8.0%)	2.68 (1.25–5.75)	2.19 (0.99–4.88)
Treatment‐induced clearance	426 (37%)	31 (7.3%)	2.43 (1.25–4.71)	2.03 (1.02–4.03)
Current infection, previously treated	44 (4%)	4 (9.1%)	3.09 (0.96–9.93)	3.28 (0.97–11.14)
Current infection, not previously treated	64 (5%)	9 (14.1%)	5.06 (2.07–12.39)	4.60 (1.80–11.75)

In adjusted analyses, factors associated with advanced chronic liver disease were older age (vs. age ≥ 50; age 30–39: aOR 0.38, 95% CI: 0.16–0.89; age 40–49: aOR 0.51, 95% CI: 0.30–0.88), women (vs. men: aOR 1.82, 95% CI: 1.07–3.09), injecting recency (vs. injected > 12 months ago; within 1–12 months: aOR 0.56, 95% CI 0.35–0.90; within last month < daily: aOR 0.47, 95% CI: 0.30–0.72; within last month ≥ daily: aOR 0.54, 95% CI 0.35–0.84), and HCV status (vs. uninfected; spontaneous clearance: aOR 2.19, 95% CI: 0.99–4.88; treatment‐induced clearance: aOR: 2.03, 95% CI: 1.02–4.03; currently infected and no previous treatment: aOR 4.60 95% CI: 1.80–11.75) (Table 3).

### 3.6 Change in Advanced Chronic Liver Disease by HCV Infection Status at First Visit

Among participants with a valid LSM in more than one wave (*n* = 341, including 42 in all three waves). Median duration between LSM was 33 months. Characteristics of those with and without LSM in more than one wave are outlined in Table S2. Change in LSM and advanced chronic liver disease was evaluated by HCV infection status at first visit. Among participants uninfected at first visit (*n* = 68), median LSM was 4.8 and 5.0 kPa at first and second visits, respectively, and advanced chronic liver disease 6% (*n* = 4) and 1% (*n* = 1) (Figure 2). Among participants with HCV clearance (spontaneous and treatment‐induced; *n* = 196), median LSM was 5.3 and 5.2 kPa at first and second visits, respectively, and advanced chronic liver disease 9% (*n* = 17) and 8% (*n* = 16). Among participants with current HCV infection at first visit (*n* = 64), median LSM declined from 5.9 kPa at first visit to 5.4 kPa at second visit, and advanced chronic liver disease from 14% (*n* = 9) to 5% (*n* = 3). Among participants with LSM in more than one wave and current HCV infection at first and second visit (*n* = 20), median LSM increased from 5.5 kPa at first visit to 6.6 kPa at second visit, and advanced chronic liver disease was 5% and 5% (*n* = 1). Among participants with LSM in more than one wave and current HCV infection at first visit and no current HCV infection at second visit (*n* = 44), median LSM declined from 5.9 kPa at first visit to 5.3 kPa at second visit, and advanced chronic liver disease from 19% (*n* = 8) to 5% (*n* = 2).

**FIGURE 2:**
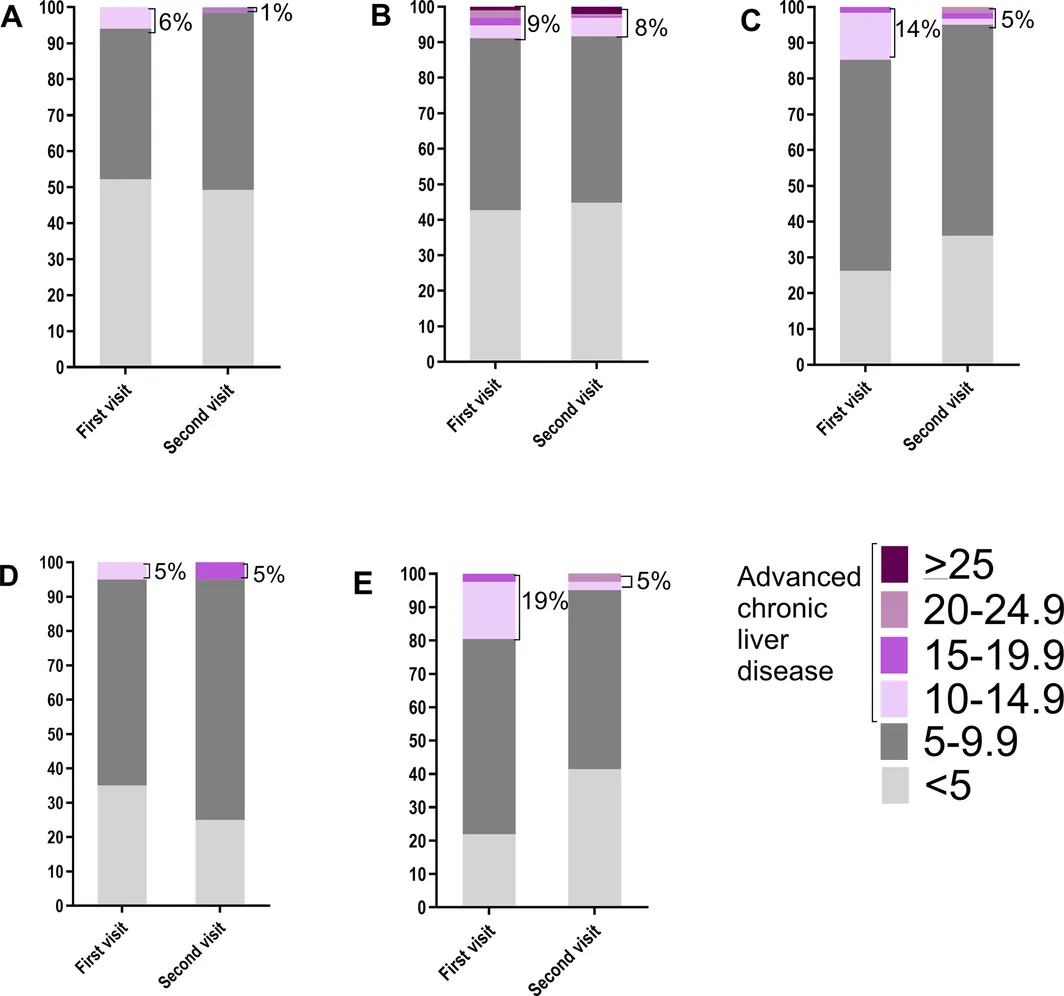
(A) Liver disease stage among people never infected with HCV at first visit (*n* = 68). (B) Liver disease stage among people reporting spontaneous clearance or treatment‐induced clearance at first visit (*n* = 196). (C) Liver disease stage among people with current HCV infection at first visit (*n* = 64). (D) Liver disease stage among people with current HCV infection at both first and second visit (*n* = 20). (E) Liver disease stage among people with current HCV infection at first visit only (*n* = 44).

## 4 Discussion

The curative success of DAA therapies is clear, but characterization of their wider health benefits including impact on liver disease burden in different populations is crucial. Our study, based on cross‐sectional liver disease evaluation repeated in three time periods, indicates a favourable impact of unrestricted access to DAA therapies with high treatment uptake on the liver health of people who inject drugs. The reduction in advanced chronic liver disease from 12.0% in 2018–2019 to 7.9% in 2019–2021 and 6.4% in 2023–2025 should translate to reduced liver‐related morbidity and mortality in this population. The modest decrease between 2019–2021 and 2023–2025 and continued higher prevalence in people with current HCV infection suggests further interventions are needed to improve liver health among people who inject drugs. Nonetheless, the results highlight successes in Australia's HCV response.

Key factors associated with advanced chronic liver disease were age, HCV status, and recruitment wave. Risk of advanced chronic liver disease increased with age, even when adjusted for HCV status and recruitment wave. Older age is clearly associated with advanced liver disease [22, 23, 24], related to longer HCV infection [25] and other liver disease co‐morbidities. Metabolic dysfunction‐associated steatotic liver disease increases with age [26] and may become a major contributor to liver disease among people who inject drugs as HCV prevalence declines. To assess this outcome, ongoing enrolment into ETHOS Engage in Wave 4 includes collection of data on body mass index (BMI) and the controlled attenuation parameter (CAP) score within hepatic elastography assessment. A CAP score above 290 is consistent with severe hepatic steatosis. As many community‐based clinical settings do not have access to hepatic elastography, the low prevalence of advanced chronic liver disease among those aged less than 40 years could be considered together with other factors, such as alcohol consumption and BMI, to determine whether referral for LSM is required.

There was a stepped increase in advanced chronic liver disease risk according to HCV status, from never infected to treatment‐induced clearance then current infection. Many participants with treatment‐induced clearance will have had chronic HCV for sufficient time to progress to advanced chronic liver disease prior to DAA therapy. Our findings do support other studies demonstrating liver disease regression following HCV cure [23, 27]. The impact of increased DAA therapy and associated lower HCV prevalence translated to reduced LSM score and advanced chronic liver disease from Wave 1 to Wave 2. Additionally, the longitudinal analysis found that among people with current HCV infection at first visit but not second, prevalence of advanced chronic liver disease declined from 19% to 5%. Conversely, for people who had current HCV infection at both visits, prevalence of advanced chronic liver disease remained at 5%, with disease progression from 10–14.9 kPa to 15 kPa or higher (the cut off point for compensated advanced chronic liver disease) [20].

Of note, more recent injecting drug use was associated with a lower risk of advanced chronic liver disease, following adjustment for age, HCV status, and alcohol consumption. A potential explanation is confounding, due to the absence of variables such as BMI and liver steatosis. This finding highlights the relative equity of access to DAA therapy, with people who currently inject drugs having similar DAA therapy uptake and current HCV prevalence to those who injected in the past. The lower risk of advanced chronic liver disease among those with more recent injecting drug use emphasizes the crucial role of BMI assessment and CAP score to investigate potential confounding.

In this study, alcohol misuse was associated with advanced chronic liver disease in the sub‐analysis of people ever HCV infected. This demonstrates the additive effect of HCV and alcohol misuse on liver health. The proportion reporting severe alcohol misuse (11%) aligns with a study in Melbourne, Australia [28], and is lower than studies in Baltimore, United States [29], and Montreal, Canada [30]. In the total sample, the prevalence of advanced chronic liver disease among those with severe alcohol misuse was comparable with the prevalence among people with low alcohol consumption. This again highlights the crucial role of HCV as a determining factor in the liver health of people who inject drugs.

This analysis highlights several implications for research and clinical practice. Collecting more data on factors relevant to liver health (alcohol consumption, BMI, waist circumference, CAP score) is important to inform an algorithm to support clinical decision‐making and to identify people at risk of advanced chronic liver disease. Despite the curative success of DAA therapies, there is still a need to investigate other interventions to improve liver health in this population. New therapies such as semaglutide have demonstrated potential for the management of liver disease and could be helpful to reduce the burden of liver disease among people who inject drugs. Finally, the collection of longitudinal data on liver health among people who inject drugs is important to understand the long‐term impact of progress toward HCV elimination.

There were several limitations to this study. Not everyone enrolled in ETHOS Engage had a valid LSM, and those with invalid results may have a higher BMI. ETHOS Engage recruitment was opportunistic, and most recruitment sites provided OAT, potentially underrepresenting the wider population of people who have a history of injecting drug use. We did not collect HCV genotype. Previous data suggest a high proportion of genotype 3 in Australia [31], which may be associated with liver disease progression [32] even after successful DAA therapy. The absence of an XL probe in Wave 1 may have slightly inflated LSM for those who required an XL probe but were scanned with an M probe [33]. We did not collect information on all factors relevant to liver health, including weight and BMI, which may have contributed toward confounding. Finally, a minority of participants had longitudinal liver stiffness data, limiting this aspect of the study.

In conclusion, this study demonstrates declining advanced liver disease among people who inject drugs in the era of DAA therapies for HCV. Further strategies to improve liver health among people who inject drugs include improving access to HCV prevention, testing, and treatment, and addressing other liver disease co‐morbidities including metabolic dysfunction‐associated steatotic liver disease.

## Author Contributions

**Anna Conway:** writing – original draft, writing – review and editing, methodology, formal analysis. **Heather Valerio:** writing – original draft, writing – review and editing, methodology, formal analysis. **Jessie Payne:** writing – review and editing, project administration. **David Silk:** writing – review and editing, project administration. **Alison D. Marshall:** conceptualization, writing – review and editing. **Marianne Martinello:** writing – review and editing. **Shane Tillakeratne:** data curation, writing – review and editing. **Putu Novi Arfirsta Dharmayani:** formal analysis. **Andrew Milat:** writing – review and editing. **Janaki Amin:** conceptualization, methodology, writing – review and editing. **Phillip Read:** resources, writing – review and editing. **Louisa Degenhardt:** writing – review and editing. **Annabelle Stevens:** writing – review and editing. **Jeremy Hayllar:** resources, writing – review and editing. **Michael Christmass:** resources, writing – review and editing. **Victoria Cock:** resources, writing – review and editing. **Jessica Doumany:** writing – review and editing. **Jason Grebely:** conceptualization, supervision, funding acquisition, methodology writing – review and editing. **Gregory J. Dore:** conceptualization, supervision, methodology, writing – review and editing.

## Funding

The Enhancing Treatment of Hepatitis C in Opioid Substitution Settings (ETHOS) Engage study is funded by the National Health & Medical Research Council (NHMRC) Partnership project grants (1103165, 2006282), and funding from New South Wales Health and Cepheid. Cepheid was not involved in the study design, methodology, and writing of this manuscript. The opinions expressed in the paper are those of the authors and do not necessarily represent those of Cepheid. This study was also supported in part by a research grant from the Australian Department of Health. The Kirby Institute is also funded by the Australian Department of Health. The views expressed in this publication do not necessarily represent the position of the Australian Government.

The contents of the published material are solely the responsibility of the individual authors and do not reflect the views of NHMRC. For the purposes of open access, the author has applied a CC BY public copyright license to any Author Accepted Manuscript version arising from this submission. The Kirby Institute is funded by the Australian Government Department of Health and Ageing. The views expressed in this publication do not necessarily represent the position of the Australian Government.

L.D. is supported by a National Institute of Health grant (R01DA1104470). A.D.M. (2026888), M.M. (2034418), L.D. (2016825), G.J.D. (2008276) and J.G. (1176131, 2034002) are supported by NHMRC Investigator Grants.

## Conflicts of Interest

H.V. has received honorarium from Gilead Sciences. A.D.M. has received research grants from Gilead Sciences. M.M. has consulted/advised for Abbvie. J.G. reports grants from Camurus, grants from Cepheid, grants from Hologic, grants from Indivior, grants from Merck, during the conduct of the study; grants and personal fees from Abbvie, grants and personal fees from Gilead Sciences, grants and personal fees from Merck, grants and personal fees from Cepheid, outside the submitted work. G.J.D. has received research grants from AbbVie and Gilead. L.D. has received investigator‐initiated untied educational grants for studies of opioid medications in Australia from Indivior, Mundipharma and Seqirus. All remaining authors have no potential conflicts to declare.

## Supporting information


**Table S1:** Factors associated with advanced fibrosis among unique ETHOS Engage participants with available fibrosis score and ever HCV infected.
**Table S2:** Characteristics of those with and without liver stiffness measure (LSM) in more than one wave.

## Data Availability

This publication involved information collected from consenting individuals. Data used for this research cannot be deposited on servers other than those approved by Ethics Committees. This publication has used highly sensitive health information through the collection of survey data. All identifying information, including full name, has been anonymized under strict privacy regulations. Except in the form of conclusions drawn from the data, researchers do not have permission to disclose any data to any person other than those authorized for the research project.
