# Supplementary material for: Reductions in Advanced Chronic Liver Disease Among People Who Inject Drugs: The ETHOS Engage Study
Source: Liver Int. 2026 Oct 30;46(12):e70940. doi: 10.1111/liv.70940 (PMC13720508; doi:10.1111/liv.70940)
Supplement: Supplementary file 1 [file LIV-46-0-s001.docx]

Supplementary Table 1: Factors associated with advanced fibrosis among unique ETHOS Engage participants with available fibrosis score and ever HCV infected

| Characteristic | | Total known fibrosis result, n (% col) | Advanced fibrosis, n (% row) | OR (95% CI) | aOR (95% CI) |
| --- | --- | --- | --- | --- | --- |
| **Total** | | 2,023 (100%) | 234 (11.6%) |  |  |
| Age at enrolment | 18-29 | 95 (5%) | 2 (2.1%) | **0.11 (0.03-0.44)** | **0.10 (0.02-0.42)** |
|  | 30-39 | 473 (23%) | 40 (8.5%) | **0.46 (0.31-0.67)** | **0.41 (0.27-0.61)** |
|  | 40-49 | 810 (40%) | 84 (10.4%) | **0.58 (0.42-0.78)** | **0.55 (0.40-0.76)** |
|  | >=50 | 645 (32%) | 108 (16.7%) |  |  |
| Gender | Men | 1,376 (68%) | 167 (12.1%) |  |  |
|  | Women | 631 (31%) | 65 (10.3%) | 0.83 (0.61-1.13) |  |
|  | Other | 16 (1%) | 2 (12.5%) | 1.03 (0.23-4.59) |  |
| Aboriginal or Torres Strait Islander | No | 1,508 (75%) | 185 (12.3%) |  |  |
|  | Yes | 515 (25%) | 49 (9.5%) | 0.75 (0.54-1.05) | 0.90 (0.64-1.28) |
| Homeless | No | 1,795 (89%) | 218 (12.1%) |  |  |
|  | Yes | 228 (11%) | 16 (7.0%) | **0.55 (0.32-0.93)** | 0.59 (0.34-1.02) |
| OAT status | Never | 181 (9%) | 23 (12.7%) |  |  |
|  | Past | 302 (15%) | 31 (10.3%) | 0.79 (0.44-1.40) |  |
|  | Current | 1,540 (76%) | 180 (11.7%) | 0.91 (0.57-1.45) |  |
| Incarceration history | Never | 478 (24%) | 60 (12.6%) |  |  |
|  | History only | 1,141 (56%) | 141 (12.4%) | 0.98 (0.71-1.36) | 1.02 (0.73-1.43) |
|  | Recent | 404 (20%) | 33 (8.2%) | **0.62 (0.40-0.97)** | 0.80 (0.50-1.29) |
| Recency of injecting | >12 months | 245 (12%) | 44 (18.0%) |  |  |
|  | Within 1-12 months | 382 (19%) | 46 (12.0%) | 0.63 (0.40-0.98) | **0.56 (0.35-0.90)** |
|  | Within last month, <daily | 684 (34%) | 70 (10.2%) | 0.52 (0.35-0.78) | **0.47 (0.30-0.72)** |
|  | Within last month, ≥daily | 712 (35%) | 74 (10.4%) | **0.53 (0.35-0.79)** | **0.54 (0.35-0.84)** |
| AUDIT-C alcohol use | Low/no use | 1,249 (62%) | 134 (10.7%) |  |  |
|  | Mild misuse | 202 (10%) | 21 (10.4%) | 0.97 (0.59-1.57) | 1.05 (0.64-1.74) |
|  | Moderate misuse | 354 (18%) | 46 (13.0%) | 1.24 (0.87-1.78) | 1.33 (0.92-1.93) |
|  | Severe misuse | 202 (10%) | 31 (15.3%) | 1.51 (0.99-2.30) | **1.66 (1.07-2.59)** |
| HCV status | Spontaneous clearance | 498 (25%) | 40 (8.0%) |  |  |
|  | Treatment-induced clearance | 979 (48%) | 97 (9.9%) | 1.26 (0.86-1.85) | 1.19 (0.80-1.76) |
|  | Current infection, previously treated | 127 (6%) | 25 (19.7%) | 2.81 (1.63-4.83) | **3.45 (1.96-6.05)** |
|  | Current infection, not previously treated | 419 (21%) | 72 (17.2%) | 2.38 (1.58-3.58) | **2.49 (1.62-3.83)** |
| Recruitment Wave | Wave 1 (2018-2019) | 897 (44%) | 130 (14.5%) |  |  |
|  | Wave 2 (2019-2021) | 548 (27%) | 53 (9.7%) | **0.63 (0.45-0.89)** | **0.66 (0.46-0.94)** |
|  | Wave 3 (2023-2025) | 578 (29%) | 51 (8.8%) | **0.57 (0.41-0.80)** | **0.61 (0.43-0.88)** |

Supplementary Table 2. Characteristics of those with and without liver stiffness measure (LSM) in more than one wave

| Characteristic | | LSM not available in more than one wave, n (% col) | LSM available in more than one wave, n (% col) |
| --- | --- | --- | --- |
| **Total** | | 2785 (100%) | 341 (100%) |
| Age at enrolment | 18-29 | 259 (9%) | 20 (6%) |
|  | 30-39 | 703 (25%) | 89 (26%) |
|  | 40-49 | 1044 (37%) | 138 (40%) |
|  | >=50 | 779 (28%) | 94 (28%) |
| Gender | Men | 1881 (68%) | 225 (66%) |
|  | Women | 880 (32%) | 114 (33%) |
|  | Other | 24 (1%) | 2 (1%) |
| Aboriginal or Torres Strait Islander | No | 2089 (75%) | 257 (75%) |
|  | Yes | 696 (25%) | 84 (25%) |
| Homeless | No | 2440 (88%) | 316 (93%) |
|  | Yes | 345 (12%) | 25 (7%) |
| OAT status | Never | 498 (18%) | 14 (4%) |
|  | Past | 435 (16%) | 38 (11%) |
|  | Current | 1852 (66%) | 289 (85%) |
| Incarceration history | Never | 900 (32%) | 89 (26%) |
|  | History only | 1374 (49%) | 191 (56%) |
|  | Recent | 511 (18%) | 61 (18%) |
| Recency of injecting | >12 months | 332 (12%) | 50 (15%) |
|  | Within 1-12 months | 572 (21%) | 66 (19%) |
|  | Within last month, <daily | 909 (33%) | 130 (38%) |
|  | Within last month, ≥daily | 972 (35%) | 95 (28%) |
| AUDIT-C alcohol use | Low/no use | 1651 (59%) | 230 (67%) |
|  | Mild misuse | 269 (10%) | 34 (10%) |
|  | Moderate misuse | 533 (19%) | 44 (13%) |
|  | Severe misuse | 308 (11%) | 31 (9%) |
| HCV status at first visit | Uninfected | 927 (33%) | 68 (20%) |
|  | Spontaneous clearance | 437 (16%) | 67 (20%) |
|  | Treatment-induced clearance | 866 (31%) | 129 (38%) |
|  | Current infection, previously treated | 111 (4%) | 17 (5%) |
|  | Current infection, not previously treated | 373 (13%) | 47 (14%) |
| First enrolled in | Wave 1 (2018-2019) | 1025 (37%) | 262 (77%) |
|  | Wave 2 (2019-2021) | 781 (28%) | 79 (23%) |
|  | Wave 3 (2023-2025) | 979 (35%) | 0 (%) |
